# The megaherbivore gap after the non-avian dinosaur extinctions modified trait evolution and diversification of tropical palms

**DOI:** 10.1098/rspb.2021.2633

**Published:** 2022-04-13

**Authors:** Renske E. Onstein, W. Daniel Kissling, H. Peter Linder

**Affiliations:** ^1^ Evolution and Adaptation, German Centre for Integrative Biodiversity Research (iDiv) Halle–Jena–Leipzig, Leipzig 04103 Germany; ^2^ Institute for Biodiversity and Ecosystem Dynamics (IBED), University of Amsterdam, Amsterdam, Noord-Holland, The Netherlands; ^3^ Department of Systematic and Evolutionary Botany, University of Zurich, Zurich, ZH Switzerland

**Keywords:** adaptation, defensive organs, extinction, plant functional trait, herbivory, Palmae

## Abstract

The Cretaceous–Palaeogene (K-Pg) extinction of the non-avian dinosaurs (66 Ma) led to a 25 million year gap of megaherbivores (>1000 kg) before the evolution of megaherbivorous mammals in the Late Eocene (40 Ma). The botanical consequences of this ‘Palaeocene megaherbivore gap’ (PMHG) remain poorly explored. We hypothesize that the absence of megaherbivores should result in changes in the diversification and trait evolution of associated plant lineages. We used phylogenetic time- and trait-dependent diversification models with palms (Arecaceae) and show that the PMHG was characterized by speciation slowdowns, decreased evolution of armature and increased evolution of megafaunal (≥4 cm) fruits. This suggests that the absence of browsing by megaherbivores during the PMHG may have led to a loss of defence traits, but the absence of megaherbivorous seed dispersers did not lead to a loss of megafaunal fruits. Instead, increases in PMHG fruit sizes may be explained by simultaneously rising temperatures, rainforest expansion, and the subsequent radiation of seed-dispersing birds and mammals. We show that the profound impact of the PMHG on plant diversification can be detected even with the overwriting of adaptations by the subsequent Late Eocene opening up of megaherbivore-associated ecological opportunities. Our study provides a quantitative, comparative framework to assess diversification and adaptation during one of the most enigmatic periods in angiosperm history.

## 1. Introduction

Megaherbivores (>1000 kg) have impacted terrestrial ecosystems at least since the Jurassic, approximately 201 million years ago (Ma) [1], and non-avian dinosaurs occupied this megaherbivore niche for most of the Mesozoic (*ca* 252–66 Ma). With the Cretaceous–Palaeogene (K-Pg) extinction of non-avian dinosaurs *ca* 66 Ma, terrestrial ecosystems faced a 25-million-year (Myr) ‘megaherbivore gap’. Although several lineages of mammals survived the K-Pg extinction event, such as the placental mammals (Placentalia), these were small, rat-sized animals. Subsequently, mammal body size increased gradually, reaching sizes comparable to large non-avian dinosaurs (i.e. of at least 1000 kg) only in the Late Eocene (*ca* 40 Ma) [2,3]. These early mammalian beasts included herbivorous Dinocerata (hoofed mammals with horns, extinct) and Perissodactyla (odd-toed ungulates, e.g. horses, rhinos, tapirs and several extinct lineages) [2]. Megaherbivores roamed the world until the Late Pleistocene and Holocene, during which most megaherbivores and at least 65% of other large-bodied animal genera (>44 kg) went extinct [4]. Although the effects of the relatively recent Pleistocene extinctions on ecosystems have been studied to some extent (e.g. [5,6]), the botanical consequences of the Palaeocene megaherbivore gap (PMHG) remain enigmatic.

The PMHG probably changed the selective regime for angiosperms [7], as ecological studies from recent time scales have shown that megaherbivores are important ecosystem modifiers with a remarkable impact on vegetation [5,8]. Most of the evidence for this comes from our understanding of extant megaherbivores such as African elephants (*Loxodonta africana*), which can break trees and so create an open shrubland, allowing light to penetrate to the ground layer and facilitating the growth of grasses and the spread of fires [8–10]. Furthermore, megaherbivores can act as effective dispersers of large, ‘megafaunal’ (>4 cm) fruits [5,6], and provide a selective advantage to spiny defences or lack of palatability [10,11]. Angiosperms are therefore expected to show numerous adaptations to the presence of, and potential interaction with, megaherbivores. Nevertheless, megafaunal fruits and spiny defences do not exclude interactions with smaller-bodied dispersers and grazers [11,12].

A world with megaherbivores has been the norm rather than the exception in angiosperm evolution, as approximately 95 Myr of their 120 Myr history has been shared with megaherbivores. Although the evidence for non-avian dinosaur-angiosperm interactions is generally poor [13], the remains of seeds, fruits, cuticles and other silicified plant tissues in dinosaur coprolites suggest that trophic interactions such as herbivory and frugivory between dinosaurs and angiosperms existed [14–16]. Similarly, the evolution of some sort of physical defence in many plants in the Cretaceous may have been in response to non-avian dinosaur browsing, especially when making the analogy to present-day ecosystems and functional plant responses to browsing [14]. It is therefore plausible that the evolution of angiosperms—including diversification dynamics and trait evolution—was initially impacted by megaherbivorous, non-avian dinosaurs, and thus dates back to the Cretaceous [14,17,18].

Theoretically, the changed selective regime in the PMHG is expected to have resulted in changes in 'ecological opportunities' for angiosperm diversification and trait evolution (comparable to Simpson's ‘adaptive zone’ [19]). For instance, the lack of ecological opportunities and selection pressures exerted by megaherbivores may have led to diversification and trait evolution rate shifts around the onset and termination of the PMHG, such as speciation slowdowns of lineages with large fruits or defence traits (e.g. spines) and an overall loss of these traits across lineages. However, several other environmental parameters, in addition to the absence of megaherbivores, were different during the PMHG compared to the preceding and subsequent periods. For instance, the PMHG also coincided with a changed climatic regime: increasing Late Palaeocene temperatures followed by gradual cooling after the Early Eocene Climatic Optimum from *ca* 50 Ma onwards [20]. These Late Palaeocene/Eocene ‘greenhouse’ climates could have facilitated the geographical expansion of rainforests and other wet, seasonal/monsoonal forests into higher latitudes, with associated phenotypic changes such as increases in seed size and animal seed dispersal [21–24]. Furthermore, the asteroid impact thought to have caused the non-avian dinosaur extinctions may have led to diversification shifts through increases in extinction rates of competitors or turnover of functional types and vegetation [25–28]. Hence, it remains unclear to what extent changes in diversification and trait evolution associated with the lack of megaherbivores during the PMHG can be detected.

Here, we aim to quantify the potential relationship between the PMHG selective regime and angiosperm diversification and trait evolution using a macroevolutionary comparative approach. Although using molecular phylogenetic data to evaluate deep-time dynamics is challenging, simulations have shown that diversification changes on phylogenies can be accurately inferred under different simulation scenarios [29]. We integrate phylogenetic, fossil and trait data, and focus on the palm family (Arecaceae), a relatively old angiosperm clade (crown node age approximately 110 Ma) typically occurring in tropical rainforests. Palms comprise approximately 2500 extant species and show a spectacular diversity of fruit sizes (from 0.3 to 45 cm in length, most of them dispersed by animals including megafauna) and armature (e.g. spiny leaves and/or stems) [30–33]. Furthermore, palms co-occurred with dinosaurs in the Cretaceous and may have been important dinosaur food [34], and there are instances of seed-like structures of palms in dinosaurian coprolites from the Cretaceous Lameta Formation in India [15,16]. Although the early mammalian megaherbivores were probably non-selective grazers and browsers with occasional fruit in their diet [7], some of their extant descendants often heavily rely on palm fruits [35]. We hypothesize that trait states associated with megaherbivores, such as large, megafaunal fruits and armature (i.e. leaf or stem spines, hooks or prickles) appear in the Cretaceous, in concordance with the presence of megaherbivorous dinosaurs (H1). We further expect speciation slowdowns during the PMHG due to a loss of megaherbivore-created ecological opportunities (H2), and that large fruits and armature have been lost or evolved less frequently during the PMHG due to the absence of megaherbivore selection pressures (H3). We test these hypotheses using ancestral state reconstruction and time- and trait-dependent diversification rate models, with specific predictions for megaherbivore and non-megaherbivore palm trait states (table 1).

**Table 1.** Hypotheses and predictions on trait evolution and diversification during the megaherbivore gap. Predictions for lineages with trait states associated with megaherbivores (large/megafaunal fruits, armature) or not (opposite trait states: small fruits and no armature) are defined to provide a comparative framework for the analysis. Support for these predictions from our analyses of palms (Arecaceae) is provided in the last two columns. PMHG = Palaeocene megaherbivore gap.

hypothesis	prediction for megaherbivore traits (large fruits, armature)	prediction for the opposite trait states (small fruits, no armature)	support from megafaunal fruit evolution	support from armature evolution
H1: trait states associated with megaherbivores (e.g. large fruits, armature) appear in the Cretaceous (>66 Ma) in concordance with the presence of megaherbivorous, non-avian dinosaurs	ancestral trait reconstructions should indicate a high probability of the presence of megaherbivore traits in Cretaceous palm lineages	ancestral trait reconstructions should indicate a low probability of the presence of non-megaherbivore traits in Cretaceous palm lineages	supported (figure 1)	supported (figure 2)
H2: speciation rates of lineages with megaherbivore traits (e.g. large fruits, armature) are higher in the presence of megaherbivores (i.e. Cretaceous and Late Eocene-to-present) than during the PMHG (66–40 Ma), due to ecological opportunities created by megaherbivores	lower speciation rates of lineages with megaherbivore traits during the PMHG compared to the megaherbivore periods	equal or higher speciation rates of lineages with non- megaherbivore traits during the PMHG compared to the megaherbivore periods	not supported—rates of lineages with megafaunal fruits are constant through time (figure 3a), whereas small fruits show lower rates during the PMHG (electronic supplementary material, figure S1a)	partly supported—rates of lineages with (figures 3b,c) or without (electronic supplementary material, figures S1b,S1c) armature are lower during the PMHG
H3: trait states associated with megaherbivores (e.g. large fruits, armature) appear more frequently in presence of megaherbivores (i.e. Cretaceous and Late Eocene-to-present) than during the PMHG (66–40 Ma)	lower transition rates to megafaunal fruits and armature during the PMHG compared to the megaherbivore periods	equal or higher transition rates to small fruits and armature loss during the PMHG compared to the megaherbivore periods	not supported—rates of megafaunal fruit evolution are higher during the PMHG (figure 3d), whereas small fruit evolution is lower during the PMHG (electronic supplementary material, figure S1d)	partly supported—rates of armature evolution are lower (figure 3e) or higher (stem armature) (figure 3f) during the PMHG, whereas the loss of armature is equal (electronic supplementary material, figure S1e) or higher (stem armature) (electronic supplementary material, figure S1f) during the PMHG

## 2. Material and methods

### (a) Palm trait data

Fruit lengths and presence/absence of armature were taken from the PalmTraits 1.0 database [31] and updated to the latest palm taxonomy, matching the phylogenetic data (2539 accepted palm species). As binary data are required for the diversification rate analyses, we classified each species for fruit length (available for *n* = 2054 species, 81% of species) as either having small fruits (<4 cm in length, *n* = 1550 species, 87%) or large, megafaunal fruits (≥4 cm in length, *n* = 224 species, 13%) [6,36]. This dichotomy is based on the seed dispersal ecology of megafauna-dispersed plants following [36]. Armature can be expressed as stem and/or leaf spines, hooks or prickles (assessed for all species, present in *n* = 1023 species, 40%) and has been shown to be a successful defence trait against herbivory from large animals in palms [37]. Spinescent structures may also facilitate climbing. This function may be especially important in the subfamily Calamoideae, in which almost all species possess leaf armature (mainly hooks). We therefore investigated whether results focusing on any type of armature (i.e. leaf and stem combined) were consistent with results when evaluating species with stem (*n* = 205 species, 8%) and/or leaf (*n* = 1015 species, 40%) armature separately.

### (b) Palm phylogenetic data

We used phylogenetic data from an all-evidence Bayesian supertree approach, including all 2539 palm species based on a mix of species-level genetic and taxonomic information [38]. Diversification analyses were performed on a random sample of 100 phylogenetic trees from the posterior distribution to account for the effects of phylogenetic uncertainty on the results. Furthermore, we performed phylogenetic simulations to evaluate the robustness of results with respect to trait evolution and phylogeny (see electronic supplementary material, methods for details). By including all extant palm species and their traits, we maximized the number of independent evolutionary events while reducing pseudo-replication [39].

### (c) Palm fruit and seed fossil data

To infer fruit size evolution in palms (H1), we combined phylogenetic data with fruit and seed fossil data. We searched the literature for palm fossil fruits and seeds following a review of the palm fossil record by ref. [40], with additional records from more recent palm fossil assessments [41,42]. This resulted in 90 observations for which we assembled the taxonomy of the fossil and its nearest living relatives (tribe, genus and species), fossil site location, fossil age, and length and/or width of the fossil fruit or seed. Although seed size differs from fruit size, they are strongly correlated in palms [33] as most fruits bear a single or a few seeds. Seed size thus provides a minimum size estimate of fruit size for a particular taxon and time period.

### (d) Ancestral reconstructions

To assess whether megaherbivore traits (large fruits and armature) were present in the Cretaceous and thus originated in the presence of megaherbivorous dinosaurs (H1, table 1), we performed ancestral state reconstructions on the palm maximum clade credibility (MCC) phylogenetic tree. Using continuous, log-transformed fruit length data, we reconstructed ancestral states for each node (ancestor) using a maximum-likelihood approach. We re-rooted the tree at all internal nodes and computed the contrast state at the root of the tree each time, and then interpolated the states along each edge of the tree. This was done with the 'fastAnc' function in the R package ‘phytools’ [43]. Based on this reconstruction, we plotted a traitgram [44] which is a projection of the phylogenetic tree in a space defined by a continuous trait—here fruit size—on the *y*-axis, and time on the *x*-axis. This shows how fruit size ‘trait space’ has evolved over time. Within this traitgram, we visualized the seed and fruit fossil data using the midpoint of the epoch the fossil was found, to evaluate whether fossil seed and fruit size fall within the reconstructed fruit size space based on the molecular data, at that respective point in time. We additionally explored how the reconstruction was affected when including the large (approx. 17 cm long) Palaeocene fossil fruits of extinct *Nipadites* [45], by constraining the fruit size of the ancestral lineage of extant *Nypa fruticans* (subfamily Nypoideae) at the time of the fossil age. Such constraints were not possible for the other fossils due to uncertainty in the phylogenetic placement or mismatch between divergence time and fossil age [42]. Several large-fruited palm species are not currently animal-dispersed (i.e. *Cocos nucifera*, *Nypa fruticans* and *Lodoicea maldivica*). Although their fruit size may therefore not necessarily have been adaptive to selection pressures exerted by megaherbivores, removing them from the analysis would give an incomplete and possibly biased inference of fruit size evolution across ancestral palms and their descending animal-dispersed lineages. We therefore decided to include them in the analysis.

For the armature data, it was not possible to reconstruct a traitgram, as the data are binary instead of continuous. Therefore, we reconstructed ancestral leaf and stem armature (present/absent) by sampling 500 stochastic character maps and assessed the posterior probability of ancestral armature types at the internal branches and nodes of the palm phylogenetic tree. Stochastic character mapping was conducted using the make.simmap function in the R package ‘phytools’ [43].

### (e) Time-dependent diversification rate analyses

To evaluate changes in speciation (H2) and trait evolution (H3) rates in response to the changed selective regime associated with the PMHG (table 1), we applied time- and trait-dependent diversification models implemented in the R package ‘diversitree’ [46]. The time-dependent version of the binary state speciation and extinction (BiSSE) model allows the inference of speciation, extinction and transition rates of palm lineages with trait states associated with megaherbivores (e.g. megafaunal fruits and armature) compared to lineages lacking those traits (i.e. small fruits, no armature) through time. We performed our initial model selection by using cut-off values at 66 Ma and 40 Ma, creating three time slices: before 66 Ma (non-avian dinosaur megaherbivore period), 66–40 Ma (PMHG) and 40 Ma to present (mammal megaherbivore period). Although the evolution of mammalian megaherbivores was probably a more gradual process initiated *ca* 40–30 Ma, the cut-off value is a requirement for these time-dependent diversification models.

We used a step-wise model selection approach to fit up to 18 diversification rate models to the datasets (electronic supplementary material, tables S1–S4). These models contained different combinations of constrained and free parameters, namely speciation, extinction and/or transition rates were constrained to be equal for lineages with small versus megafaunal fruits, for lineages with armature versus no armature, and/or for lineages evolving in the three geological time periods, or they were allowed to differ freely. We address recent criticism on diversification inferences [47] by identifying the most likely diversification scenarios out of the set of suitable alternative scenarios, specifically designed to test our predictions (table 1; electronic supplementary material, tables S1–S4). We compared these models using likelihood-ratio tests and selected the best-fitting models given the fewest number of parameters without significantly decreasing model fit. A Bayesian Markov chain Monte Carlo (MCMC) was run for the best-fitting model for 10 000 generations on the 100 palm phylogenetic trees. We plotted the posterior distributions (95% Bayesian credibility intervals) of the parameter estimates for the speciation and transition rates during the three geological time periods. Last, we repeated the armature diversification analyses for stem and leaf armature types separately, to evaluate whether their diversification response in relation to the PMHG was equivalent.

## 3. Results

### (a) Ancestral reconstructions

**Figure 1.**
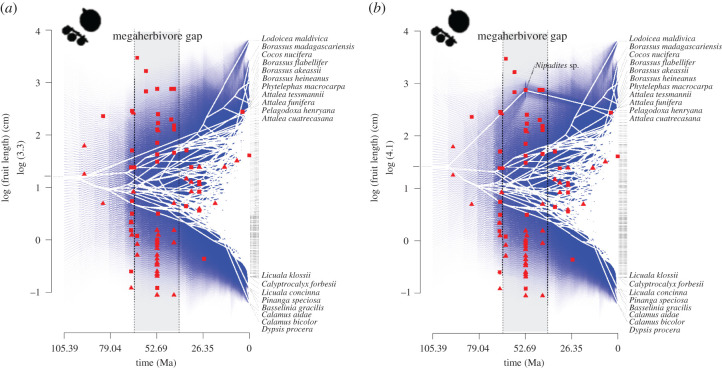
Traitgrams illustrating the evolution of log-transformed fruit lengths in palms (Arecaceae) over geological time. (*a*) Without fossil fruit size constraint; (*b*) including the constraint of fossil fruits belonging to ancestral *Nypa* palms in genus *Nipadites.* The traitgram relies on maximum-likelihood ancestral state reconstruction and is a projection of the phylogenetic tree in a space defined by fruit size (*n* = 2054 species). Confidence intervals of the maximum-likelihood estimates at the internal nodes are indicated with the blue colour. Only names of extant species with some of the largest and smallest palm fruits are indicated at the tips. Red triangles (*n* = 45) and squares (*n* = 45) illustrate, respectively, (maximum) seed and fruit fossil size, using the midpoint of the epoch the fossil was found in. This figure supports the hypothesis that Cretaceous palms already possessed large, megafaunal fruits ≥4 cm (H1, table 1). The grey bar (between stippled lines) reflects the megaherbivore gap (PMHG). (Online version in colour.)

**Figure 2.**
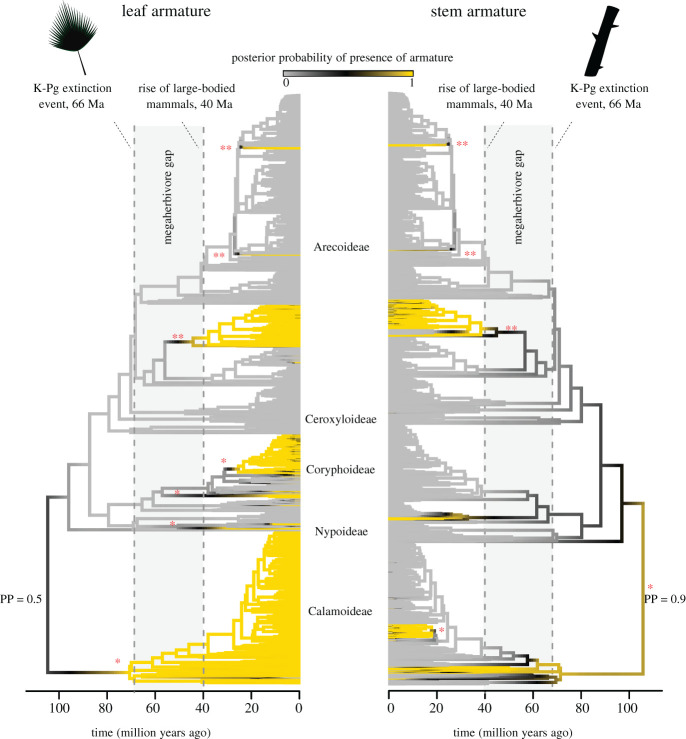
Evolution of leaf and stem armature in palms (Arecaceae). Ancestral state reconstructions were performed using stochastic character mapping. The posterior probability (PP, between 0 and 1) of ancestral lineages possessing armature is indicated with the yellow colour (i.e. more yellow means higher probability of having armature; black colour is intermediate and thus illustrates an equivocal state, grey means low probability of having armature), suggesting that Cretaceous palms probably had stem armature, but possibly not leaf armature (H1, table 1). The probability of armature at the root node is indicated. Armature evolved at least eight times independently in palms (indicated with the asterisks, simultaneous occurrences of leaf and stem armature are indicated with a double asterisk). Most origins occurred in subfamilies Coryphoideae (e.g. typical spinescent genera *Brahea, Coccothrinax, Copernicia, Corypha, Cryosophila, Hyphaene, Licuala, Livistona, Phoenix* and *Trithrinax*) and Arecoideae (e.g. genera *Aiphanes, Astrocaryum, Bactris, Butia* and *Desmoncus*) and happened from 40 Ma onwards, when megaherbivorous mammals had evolved. Subfamilies are indicated at the tips of the phylogenetic tree. The grey bar (between stippled lines) reflects the megaherbivore gap (PMHG). For the evolution of stem and leaf armature combined, see electronic supplementary material, figure S4. (Online version in colour.)

Ancestral state reconstructions supported the hypothesis (H1) that Cretaceous palm lineages probably had megafaunal fruits and that stems were covered with spines, thorns or prickles (figures 1 and 2). The traitgram without fossil constraints (figure 1*a*) shows a gradual expansion of fruit size trait space with rapid increases from the Early Oligocene (*ca* 30 Ma) onwards with the diversification of several large-fruited lineages in the Borasseae tribe (including the double coconut, *Lodoicea maldivica*) [48], whereas fruit size increase (based on molecular data only) was much slower in the Cretaceous and Early Cenozoic. The fruit and seed sizes of most fossils fall within the size ranges predicted by the molecular reconstructions based on the maximum-likelihood estimates at the internal nodes (figure 1, in blue), with the exception of some Cretaceous and Early Cenozoic fossils, mainly belonging to ancestors of *Nypa fruticans* and Cocoseae (*Cocos, Tripylocarpa* [49])*.* When including the *Nipadites* fossils in the reconstruction (figure 1*b*), fruit size trait space expands much earlier in the Cretaceous with the evolution of the *Nypa* lineage and its large fossil fruits [45].

For the evolution of armature, stochastic character maps indicated a high probability of stem armature in the ancestral palm (figure 2, i.e. a posterior probability [PP] of approximately 0.9 for stem armature), but not for leaf armature (PP = approximately 0.5). Furthermore, the first diverging lineage, subfamily Calmoideae, is characterized by armature in both leaves (all species) and stems (several lineages), and these may thus date back to the Cretaceous (at least 66 Ma, but probably older). Armature evolved at least eight times within palms (see asterisks in figure 2), in all subfamilies except for Nypoideae and Ceroxyloideae, and most of these origins happened after 40 Ma. Interestingly, in all four cases in which lineages possess both leaf and stem armature, both types of armature seem to have evolved more or less simultaneously (see double asterisks in figure 2).

### (b) Diversification dynamics of palms

Our model selection indicated that for traits associated with megaherbivores (megafaunal fruits, armature [leaf and stem combined], stem armature), a time-dependent model in which speciation, extinction and/or transition rates of palm lineages showed a significant shift during the PMHG fitted better than a constant-rate (time-independent) model (electronic supplementary material, tables S1–S3). An exception was the evolution of leaf armature, for which there was no support for temporal changes in diversification rates (electronic supplementary material, table S4). The best models for each trait indicated distinct diversification scenarios for megafaunal versus small fruits, and for armature versus no armature (electronic supplementary material, tables S1–S3).

**Figure 3.**
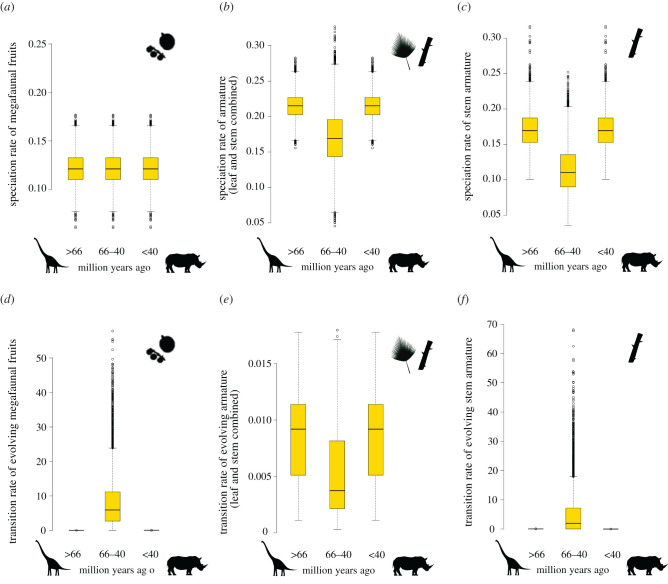
Speciation and transition rates of palm (Arecaceae) lineages with megaherbivore traits (megafaunal fruits and armature) before, during and after the megaherbivore gap (66–40 Ma). (*a*), (*b*) and (*c*) evaluate speciation slowdowns in the PMHG (H2, table 1), whereas (*d*), (*e*) and (*f*) evaluate whether trait evolution rates decreased in the PMHG (H3, table 1). (*a*) Speciation rate of megafaunal fruits; (*b*) speciation rate of armature (leaf and stem combined); (*c*) speciation rate of stem armature; (*d*) transition rate of evolving megafaunal fruits; (*e*) transition rate of evolving armature (leaf and stem combined); (*f*) transition rate of evolving stem armature. Megafaunal fruits were defined as ≥4 cm in length. Rates were inferred by fitting time-dependent binary state speciation and extinction (BiSSE) models to the phylogenetic data. BiSSE models were selected using maximum-likelihood optimization, and box-and-whiskers show 95% posterior densities of the rates resulting from Bayesian MCMC analyses over 100 phylogenetic trees, based on the diversification model with the best fit (see electronic supplementary material, tables S1–S3). Rates are given in lineages per million years. For comparison, rates of palm lineages with small fruits or those lacking armature are illustrated in electronic supplementary material, figure S1. (Online version in colour.)

Specifically, for fruit size, we did not find support for the hypothesis (H2) that speciation rates decreased for lineages with megafaunal fruits during the PMHG. Instead, the best model indicated constant rates through time (figure 3*a*), whereas speciation rates of lineages with small fruits decreased during the PMHG compared to rates before or after the PMHG (electronic supplementary material, figure S1a). For armature, we found support for the hypothesis that speciation slowed down during the PMHG for lineages with any type of armature or only stem armature (H2, figure 3*b*,*c*). However, lineages without armature also showed a speciation slowdown during the PMHG (electronic supplementary material, figure S1b and S1c). This suggests that the speciation slowdown may not exclusively depend on the presence or absence of armature.

Concerning trait evolution rates (H3), fruit size evolution did not support the hypothesis that the PMHG was associated with decreased rates of megafaunal fruit evolution. Instead, we detected increased transition rates to megafaunal fruits (from small fruits) during the PMHG, compared to rates preceding or following the PMHG (H3, figure 3*d*). In comparison, the evolution of small fruits (from megafaunal fruits) was generally low, and even slightly decreased during the PMHG compared to rates preceding or following the PMHG (electronic supplementary material, figure S1d). For armature, our results supported the hypothesis that armature evolution (leaf and stem combined) slowed down during the PMHG (H3, figure 3*e*), but not for stem armature only (figure 3*f*). For the latter, the best model indicated increased transition rates during the PMHG. Interestingly, and in comparison, the loss of armature (leaf and stem combined) remained constant through time (electronic supplementary material, figure S1e), whereas the loss of stem armature also increased during the PMHG (electronic supplementary material, figure S1f), thus supporting the hypothesis that a lack of megaherbivore selection pressures for plant defence during the PMHG may have led to a slowdown in armature evolution (table 1).

Our simulation of neutral traits on the palm phylogeny showed that most of the empirical diversification rate inferences did not result from methodological artefacts related to the palm phylogenetic tree shape (distribution of branch lengths) (compare figure 3 and electronic supplementary material, figures S1, S2 and S3). More details are provided in the electronic supplementary material, results.

## 4. Discussion

We predicted changes in selective regimes after the non-avian dinosaur extinctions during the megaherbivore gap (PMHG) from approximately 66–40 Ma, with consequences for the diversification and trait evolution of megaherbivore-associated plant lineages. We therefore studied the evolution of megaherbivore traits in the palm family, and show that some are consistent with the predictions, while others are not (table 1).

### (a) Cretaceous megaherbivore-associated plant traits

We found support for the hypothesis (H1) that large fruits and armature—typical megaherbivore traits—originated in the Cretaceous (figures 1 and 2). The evolution of these two traits is possibly correlated due to the interplay between, respectively, mutualistic and antagonistic interactions with megaherbivores [50]. Our finding is consistent with the large size of some of the Cretaceous palm fossil fruits [41,51,52], and with previous phylogenetic inferences of palm fruit size based on a binary fruit size classification (i.e. a high probability of megafaunal fruits in the ancestral palm *ca* 110 Ma [32]). This suggests that herbivorous non-avian dinosaurs may have been (occasional) seed dispersers of these large fruits [6]. Furthermore, we show that stem armature in palms dates back to the Cretaceous. This pattern is primarily driven by the early-diverging Calamoideae, which are highly spinescent, but are also typical forest climbers. It is therefore possible that spinescent structures (such as hooks in Calamoideae) initially evolved to facilitate climbing [53,54]. Subsequently, they may have either been a genetic or developmental precursor for the evolution of defence spines (e.g. longer spines on trunks), or were later put to new use, as defence against mammalian browsers, thus making them exaptive to this new function [55]. Although spiny or thorny leaves/stems or other forms of mechanical defence were probably present in Jurassic and Cretaceous seed plants [14], it remains puzzling to what extent structural defences in angiosperms initially evolved in response to dinosaur grazing or browsing.

### (b) Palm diversification in the megaherbivore gap

We also found support for the hypothesis that speciation rates slowed down in lineages with armature during the PMHG, but not in those with large fruits (H2, figure 3). However, a similar pattern of speciation slowdown was observed in lineages lacking armature (electronic supplementary material, figure S1), suggesting that speciation rates of palms were generally low during the PMHG, regardless of their megaherbivore traits. This shift in speciation rate after the non-avian dinosaur extinctions is consistent with the K-Pg mass extinction event leading to turnover of lineages as exemplified by both fossils [27] and molecular phylogenies [28]. However, it contradicts previous diversification inferences for palms, which indicated a gradual accumulation of diversity from the Early Cretaceous onwards [56]. Although Couvreur *et al*. [56] inferred net diversification rates (i.e. speciation minus extinction) whereas we focused on speciation rates, our models suggest a PMHG drop in net diversification rates as well, for all palm lineages except for those with large fruits (electronic supplementary material, figure S5).

### (c) Palm trait evolution in the megaherbivore gap

We also detected a distinct trait evolution regime during the PMHG. Specifically, we found support for the hypothesis (H3) that the absence of megaherbivore selection pressures created an environment in which armature did not provide an evolutionary advantage, illustrated by decreased innovation of armature (figure 3) and increased loss of stem armature (electronic supplementary material, figure S1) during the PMHG. Most innovations of armature in palms occurred at the end of or after the PMHG (figure 2). This is consistent with the frequent origins of spinescence across woody African savanna lineages from *ca* 40 Ma onwards, with most origins in the mid-Miocene in concordance with the radiation of medium- to large-bodied browsers (especially bovids) [11]. Our results thus suggest that spinescence in forest palms may be much older than in savanna plants (figure 2), but the explosive innovation of spinescence across angiosperms was more likely a late Cenozoic than Cretaceous phenomenon [11].

By contrast, we detected increased instead of decreased innovation of large, megafaunal fruits during the PMHG, thus rejecting H3 for fruit size. This contradicts the prediction that megafaunal palm fruits exclusively relied on herbivores of ≥1000 kg for their seed dispersal. Indeed, in contemporary ecosystems, large palm fruits of approximately 4 cm can also be dispersed by smaller-bodied frugivores, seed predators (e.g. parrots) or scatter-hoarders (e.g. rodents) [12]. Furthermore, the high rate of palm fruit size evolution during the PMHG is consistent with the large palm fossil fruits from a Palaeocene Colombian rainforest [49] and coincided with the radiation of small to medium-sized arboreal frugivorous birds and mammals [7,57] that were likely good dispersers of palm fruits [30]. Wing & Tiffney [7] argued that without the effect of large, generalized megaherbivores, competition among plants in the PMHG increased, leading to denser vegetation promoting larger plants with larger seeds. This result in palms (also see [48]) is thus consistent with the explosive increase in fruit and seed sizes and biotic dispersal across angiosperms during the PMHG [7,23]. Thus, the expansion of rainforests [21,22], due to a lack of megaherbivore disturbance and/or Late Palaeocene/Eocene greenhouse climates [20], may have triggered the changed fruit size evolution regime during the PMHG, rather than the absence of seed dispersal by megaherbivores [6].

### (d) Extinction in the megaherbivore gap

It is important to note that lineages with typical megaherbivore traits (e.g. large fruits and armature) may have suffered extinction during the PMHG, especially if they were specialized to interact with megaherbivores, and so have been largely erased from the phylogenetic record. This may have influenced our inferences of speciation and trait evolution rates, and may thus explain why we did not find exclusive support for our predictions (table 1). Potential increases in extinction rates associated with the evolution of megaherbivore traits (e.g. large fruits [32]) may also obscure inferences of speciation rates, because speciation and extinction can be correlated [58]. Finally, the palm fossil record suggests that large palm fruits were more common during the Cretaceous and PMHG than inferred from the molecular phylogenetic ancestral trait reconstruction only (figure 1). This illustrates the promising avenue of integrating fossil, phylogenetic and morphological data directly in a total-evidence phylogenetic approach to obtain more accurate estimates of the timing of past diversification and the (mis)match between fossils and molecules [59,60].

### (e) Outlook and conclusions

The PMHG after the dinosaur extinctions is unique in angiosperm evolution as it coincided with the absence of herbivores greater than 1000 kg. Here, we show that trait evolution and diversification of tropical palms were modified during this period, exemplified by decreased speciation rates, decreased innovation of leaf/stem armature and increased evolution of large fruits. Our study provides a framework to further explore how the changed selective regime in the PMHG affected the evolution of megaherbivore-associated plant and animal lineages. For example, besides armature, a release from herbivory during the PMHG could be associated with evolutionary change in lineages with plant escape, architectural or resistance traits [54]. Similarly, a loss of (specialized) megaherbivore interaction partners could have led to speciation slowdowns in dung beetles (Scarabaeidae), predators or large-bodied scavengers [61,62]. We therefore formulated several theoretical PMHG predictions (table 2) and encourage further testing of the consequences of the PMHG on plant and animal diversification. This would shed new light on evolution and adaptation during one of the most enigmatic and unique periods in angiosperm history—the megaherbivore gap.

**Table 2.** Change in selective regime during the Palaeocene megaherbivore gap (PMHG) (66–40 million years ago), and the theoretical predictions for plant and animal diversification and trait evolution. Radiations or diversification slowdowns are expected due to, respectively, a gain or loss of ecological opportunity associated with megaherbivores. These predictions largely follow the Late Quaternary megaherbivore ecology as outlined in [5].

megaherbivore ecology	PMHG selective regime shift (D = direct, I = indirect)	related plant or animal traits	PMHG diversification rate shift	PMHG trait shift
long-distance dispersal of large, ‘megafaunal’ fruits [6]	D: loss of seed dispersal	megafaunal ‘overbuilt’ fruits	slowdown in lineages with megafaunal fruits	decrease in fruit size
debarking or breaking trees, trampling seedlings, browsing or grazing [10,54]	D: herbivory release	plant escape traits (e.g. rapid growth in juvenile stage, large plant height)	slowdown in lineages with megaherbivore escape traits	loss of escape traits
		plant armature (e.g. spines, thorns)	slowdown in lineages with armature traits	loss of armature traits
		plant architecture (e.g. rhizomatous or prostate growth, divaricate branching, resprouting)	slowdown in lineages with architectural megaherbivore resistance traits	loss of architectural resistance traits
		other resistance traits (e.g. leaf palatability or latex, high wood density)	slowdown in lineages with structural or chemical megaherbivore resistance traits	loss of resistance traits
		plant defence traits (e.g. extrafloral nectaries [EFNs] associated with ant defence) [63]	slowdown in lineages with megaherbivore defence traits (e.g. EFNs)	loss of defence traits
host for (ecto- and endo-) parasites [5]	D: loss of host	parasite traits related to host resource use	slowdown in parasitic lineages specialized on megaherbivore hosts	host-switching
prey for (micro- and large carnivorous) predators [62]	D: loss of prey	predator traits related to prey resource use	slowdown in predator lineages preying on megaherbivores	diet/prey- switching, body size decrease
dung associations with dung beetles (Scarabaeidae) [61]	D: loss of dung	dung beetle diet traits and body size	slowdown in large-bodied dung beetle lineages	diet-switching between faecal types, body size decrease
large, conspicuous carcasses for scavengers [62]	D: loss of large carcasses	scavenger traits related to carcass resource use	slowdown in large-bodied scavenger lineages (e.g. vultures)	diet-switching, body size decrease, change in skull morphology, reduced niche width/specialization
other interactions with birds (e.g. parasite cleaning, megaherbivore faeces as foraging sites or nest material, etc.) [64]	D: loss of specialized interaction partner	specialized interaction traits	slowdown in megaherbivore-associated bird lineages (e.g. oxpeckers)	diet-switching, behavioural change
physically opening the vegetation, breaking trees [9]	I: dense, closed forest, loss of open habitat (unless fire replaced megaherbivores)	forest traits (e.g. tree growth form, large plant heights, seed size, biotic seed dispersal)	radiation of forest lineages, large trees, large seeds, lineages with biotic dispersal	gain of forest traits
		arborescent and understory lifestyle	radiation of arborescent or understory animals (e.g. monkeys, birds) [57]	gain of arborescent or understory lifestyle traits
		shade-tolerance	radiation of shade-tolerant, understory lineages (e.g. shade-loving grasses, lianas, epiphytes)	gain of shade-tolerance traits
		open habitat traits (e.g. narrow leaves, grasses)	slowdown in open habitat lineages (e.g. light-loving grasses, narrow-leaved lineages)	loss of open habitat traits

## Data Availability

Trait data are available from the PalmTraits 1.0 database [31]. Phylogenetic data are available from [38]. Fossil fruit and seed data and sources and R scripts to perform the analyses are available in the electronic supplementary material [65] and from the Dryad Digital Repository: https://doi.org/10.5061/dryad.f1vhhmgzt [66].
